# Supplementary material for: Towards unified AI-driven fracture mechanics: the extended deep energy method (XDEM)
Source: Nat Commun. 2026 Aug 15;17:8492. doi: 10.1038/s41467-026-76748-1 (PMC13484471; doi:10.1038/s41467-026-76748-1)
Supplement: Supplementary file 3 [file 41467_2026_76748_MOESM3_ESM.zip › pic/Results/Crack_propagation/Straight/3D_dis_force/Tension_3D/Loss_convergence_2.pdf]

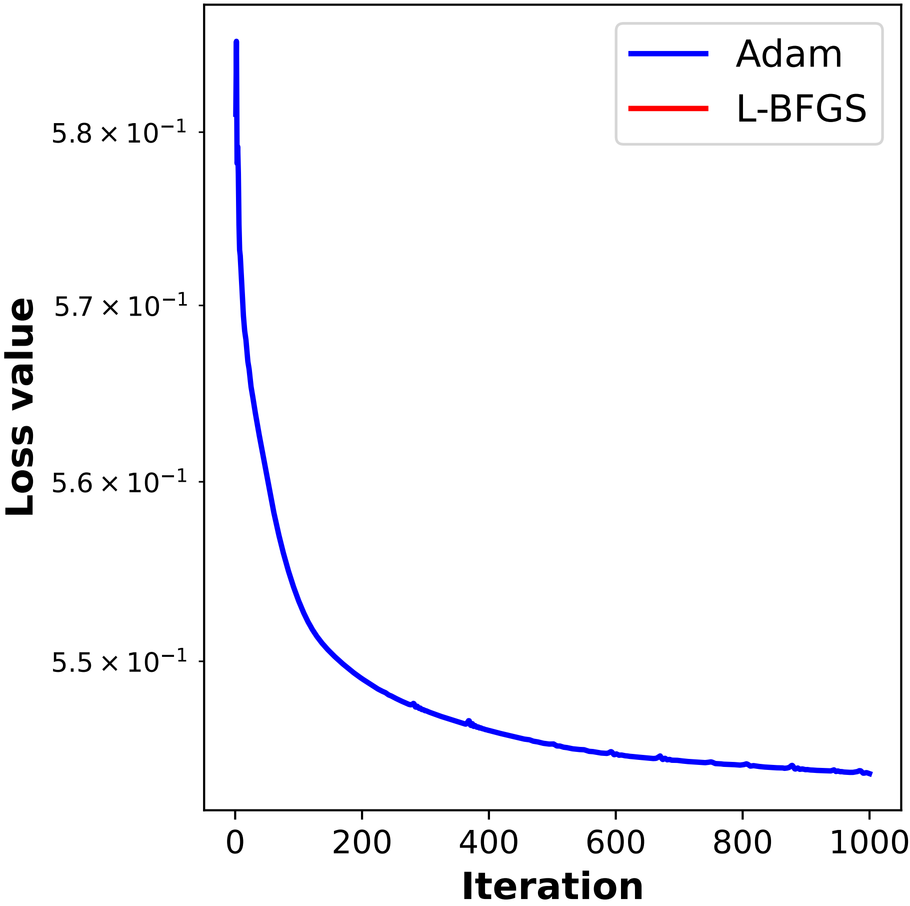

Adam
L-BFGS
5.8×10^{-1}
5.7×10^{-1}
5.6×10^{-1}
5.5×10^{-1}
Loss value
0
200
400
600
800
1000
Iteration
